# Supplementary material for: Identification and functional analysis of non-coding regulatory small RNA FenSr3 in Bacillus amyloliquefaciens LPB-18
Source: PeerJ. 2023 May 15;11:e15236. doi: 10.7717/peerj.15236 (PMC10194069; doi:10.7717/peerj.15236)
Supplement: Supplemental Information 4 [file peerj-11-15236-s004.zip › KO/CK-vs-T1_map/map04925.html]

KEGG PATHWAY: Aldosterone synthesis and secretion - Reference pathway


|  |  |
| --- | --- |
| **Aldosterone synthesis and secretion - Reference pathway** |  |

[
Pathway menu
| Organism menu
| Pathway entry
| Show description
| User data mapping
]

Aldosterone is a steroid hormone synthesized in and secreted from the outer layer of the adrenal cortex, the zona glomerulosa. Aldosterone plays an important role in the regulation of systemic blood pressure through the absorption of sodium and water. Angiotensin II (Ang II), potassium (K+) and adrenocorticotropin (ACTH) are the main extracellular stimuli which regulate aldosterone secretion. These physiological agonists all converge on two major intracellular signaling pathways: calcium (Ca2+) mobilization and an increase in cAMP production. The increase in cytosolic calcium levels activates calcium/calmodulin- dependent protein kinases (CaMK), and the increased cAMP levels stimulate the activity of cAMP-dependent protein kinase, or protein kinase A (PKA). The activated CaMK, and possibly PKA, activates transcription factors (NURR1 and NGF1B, CREB) to induce StAR and CYP11B2 expression, the early and late rate- limiting steps in aldosterone biosynthesis, respectively, thereby stimulating aldosterone secretion.

|  |  |  |
| --- | --- | --- |
| Reference pathway | 184% 150% 122% 100% 82% 67% 55% | 图片下载 |
